# Synthesis of a Vpr-Binding Derivative for Use as a Novel HIV-1 Inhibitor

**DOI:** 10.1371/journal.pone.0145573

**Published:** 2015-12-23

**Authors:** Kyoji Hagiwara, Hideki Ishii, Tomoyuki Murakami, Shin-nosuke Takeshima, Nopporn Chutiwitoonchai, Eiichi N. Kodama, Kumi Kawaji, Yasumitsu Kondoh, Kaori Honda, Hiroyuki Osada, Yasuko Tsunetsugu-Yokota, Masaaki Suzuki, Yoko Aida

**Affiliations:** 1 Viral Infectious Diseases Unit, RIKEN, 2–1 Hirosawa, Wako, Saitama 351–0198, Japan; 2 Molecular Imaging Medicinal Chemistry Laboratory, RIKEN Center for Molecular Imaging Science, 6-7-3 Minatoshima-minamimachi, Chuo-ku, Kobe, Hyogo 650–0047, Japan; 3 Division of Miyagi Community Health Promotion, Tohoku University Graduate School of Medicine, 2–1 Seiryocho, Aoba-ku, Sendai 980–8575, Japan; 4 Chemical Biology Research Group, RIKEN CSRS, 2–1 Hirosawa, Wako, Saitama 351–0198, Japan; 5 Department of Frontier Biosciences, Department of Immunology, National Institute of Infectious Diseases, Toyama, Shinjuku-ku, Tokyo 162–8640, Japan; Temple University, UNITED STATES

## Abstract

The emergence of multidrug-resistant viruses compromises the efficacy of anti-human immunodeficiency virus type 1 (HIV-1) therapy and limits treatment options. Therefore, new targets that can be used to develop novel antiviral agents need to be identified. We previously identified a potential parent compound, hematoxylin, which suppresses the nuclear import of HIV-1 via the Vpr-importin α interaction and inhibits HIV-1 replication in a Vpr-dependent manner by blocking nuclear import of the pre-integration complex. However, it was unstable. Here, we synthesized a stable derivative of hematoxylin that bound specifically and stably to Vpr and inhibited HIV-1 replication in macrophages. Furthermore, like hematoxylin, the derivative inhibited nuclear import of Vpr in an *in vitro* nuclear import assay, but had no effect on Vpr-induced G2/M phase cell cycle arrest or caspase activity. Interestingly, this derivative bound strongly to amino acid residues 54–74 within the C-terminal α-helical domain (αH3) of Vpr. These residues are highly conserved among different HIV strains, indicating that this region is a potential target for drug-resistant HIV-1 infection. Thus, we succeeded in developing a stable hematoxylin derivative that bound directly to Vpr, suggesting that specific inhibitors of the interaction between cells and viral accessory proteins may provide a new strategy for the treatment of HIV-1 infection.

## Introduction

Human immunodeficiency virus type 1 (HIV-1) is the causative agent of acquired immunodeficiency syndrome (AIDS). The most effective treatment for AIDS is chemotherapy with HIV inhibitors, of which there are four classes: those that inhibit the viral integrase, the viral protease, reverse transcriptase, or viral entry/fusion [1]. Highly active antiretroviral therapy (HAART) using a combination of protease and reverse transcriptase inhibitors suppresses HIV-1 infection, leading to a marked reduction in AIDS-related mortality [2]. Although many drugs are approved for HAART, the emergence of drug-resistant viruses severely limits their clinical effectiveness. Therefore, new classes of drugs that combat HIV-1 infection are urgently needed.

Macrophages are the cellular targets of HIV-1. These cells serve as a crucial virus reservoir and are widely distributed throughout all tissues and organs [3]. In contrast to activated CD4^+^ T lymphocytes, macrophages are resistant to the cytopathic effects of HIV and survive for long periods after infection. In addition, HIV-1 within latent-infected macrophages is not eradicated by HAART [4]. Thus, new targets for antiviral agents that inhibit HIV-1 replication in macrophages must be identified.

One such target is the HIV-1 accessory protein, Vpr, which is a 96 amino acid virion-associated protein that is conserved in all primate lentiviruses, including HIV-1 and simian immunodeficiency virus [5]. Vpr plays a key regulatory role in nuclear import of the pre-integration complex (PIC) into non-dividing cells, such as macrophages, which act as viral reservoirs [6–9]. Our previous studies show that Vpr is first targeted to the nuclear envelope and then transported to the nucleus by importin α (a process that occurs in an importin ß-independent manner) [10]. Furthermore, the interaction between Vpr and importin α is crucial, not only for the nuclear import of Vpr, but also for HIV-1 replication in macrophages [7]. Moreover, we also demonstrated that hematoxylin, which suppresses the interaction between Vpr and importin α, reduces HIV-1 replication in macrophages by blocking nuclear import of PIC [11]. However, hematoxylin was not stable under UV irradiation and was difficult to maintain. Here, we report the synthesis of a new, stable derivative of hematoxylin that inhibits the importin α-mediated nuclear import of Vpr. This derivative may play an important role in inhibiting efficient HIV-1 infection of primary macrophages. These findings may form the basis for a strategy aimed at developing a new class of anti-HIV agents.

## Results

### Synthesis of a stable hematoxylin derivative

**Fig 1:**
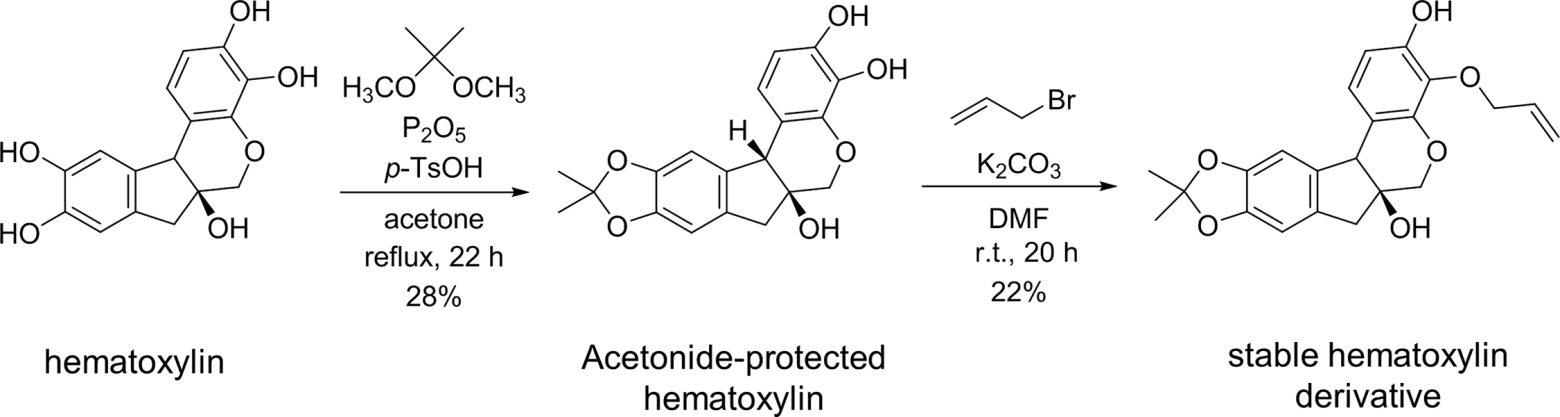
Synthesis of a stable hematoxylin derivative. Treatment of hematoxylin with 2,2-dimethoxypropane in acetone in the presence of *p*-toluene sulphonic acid and phosphorus pentoxide under reflux conditions yielded acetonide-protected hematoxylin (yield, 28%). Next, the acetonide was reacted with allyl bromide in the presence of potassium carbonate in dimethylformamide at room temperature to yield stable hematoxylin (yield, 22%). ^1^H-NMR(CDCl_3_) δ 6.97 (1 H, d, *J* = 8.5 Hz, ArH), 6.67 (1 H, s, ArH), 6.66 (1 H, d, *J* = 8.5 Hz, ArH), 6.56 (1 H, s, ArH), 6.05 (1 H, dddd, *J* = 6.3, 6.3, 10, 17 Hz, OCH_2_CHCH_2_), 5.79 (1 H, br s, ArOH), 5.34 (1 H, br dd, *J* = 1.4, 17 Hz, OCH_2_CHCH_2_), 5.24 (1 H, br d, *J* = 10 Hz, OCH_2_CHCH_2_), 4.59 (1 H, dd, *J* = 6.3, 12 Hz, OCH_2_CHCH_2_), 4.52 (1 H, dd, *J* = 6.3, 12 Hz, OCH_2_CHCH_2_), 4.11 (1 H, d, *J* = 11 Hz, CH_2_), 4.05 (1 H, s, CH), 3.85 (1 H, d, *J* = 11 Hz, CH_2_), 3.17 (1 H, d, *J* = 15 Hz, CH_2_), 2.83 (1H, d, *J* = 15 Hz, CH_2_), 2.64 (1 H, br, OH), 1.65 (3 H, s, CH_3_), 1.60 (3 H, s, CH_3_); MS (ESI pos.) *m*/*z* 383 [M+H]^+^.

A two-step process was used to synthesize a stable derivative of hematoxylin based on structure-activity relationship (SAR) studies; this derivative was synthesized because hematoxylin itself is unstable. As shown in Fig 1, hematoxylin contains four active aromatic hydroxyl groups and one aliphatic hydroxyl group. By contrast, the derivative contains only one aliphatic hydroxyl group and one aromatic hydroxyl group. The novel derivative was more stable than hematoxylin. Binding analyses were performed using surface plasmon resonance (SPR) as described below.

### SPR analysis of the binding affinity of the derivative for Vpr

**Fig 2:**
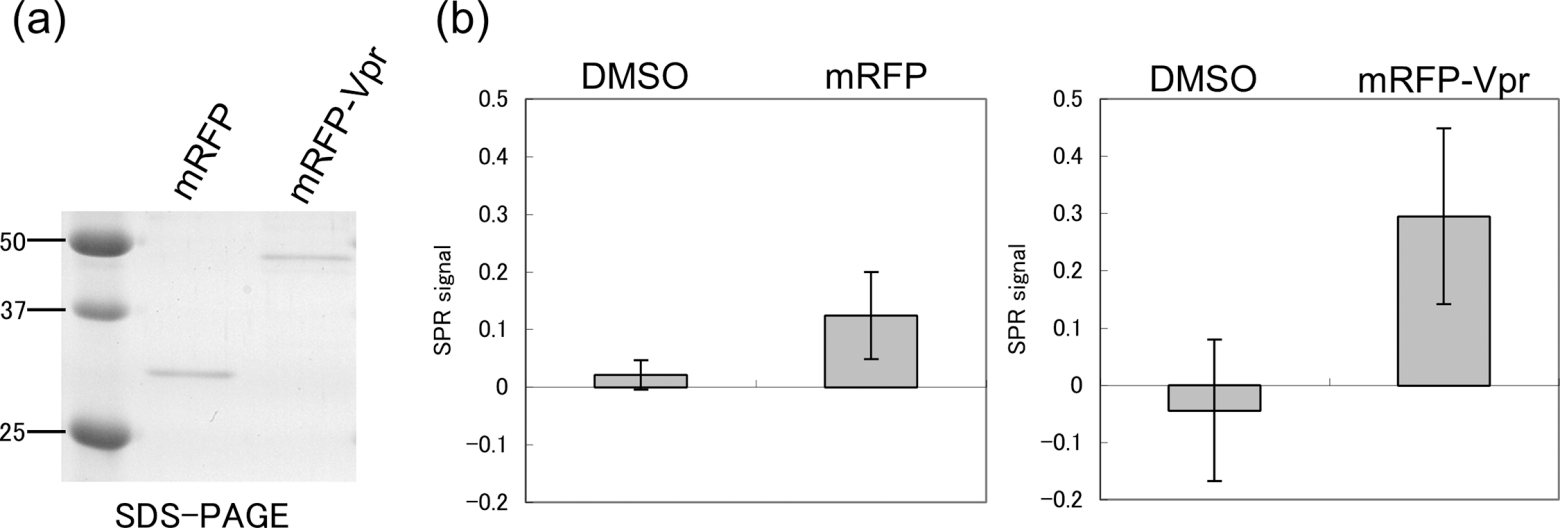
Purification of recombinant Vpr protein and binding analysis by SPR. (a) SDS-PAGE analysis of purified recombinant proteins. COS-7 cells were transfected with mammalian pCAGGS vectors encoding FLAG-mRFP (mRFP) or FLAG-mRFP-Vpr (mRFP-Vpr) and then purified on ANTI-FLAG M2 agarose beads. Proteins were separated on reducing 15% SDS-PAGE and stained with Coomassie brilliant blue. (b) SPR to determine the binding of the derivative to Vpr. The derivative was coupled to PGS and incubated with mRFP or mRFP-Vpr. DMSO was used as a negative control.

To determine the affinity of the compound for Vpr, both hematoxylin and the hematoxylin derivative were cross-linked to a photoaffinity-linker-coated gold substrate (PGS)-coated SPR chip by UV irradiation. Because the hematoxylin derivative was not damaged by UV, it was fixed stably to the PGS. By contrast, hematoxylin underwent structural changes upon exposure to UV and could not be fixed to PGS (data not shown). Next, we expressed Flag-mRFP (mRFP) and Flag-mRFP full-length Vpr (mRFP-VPR) in COS-7 cells, and observed them under a fluorescence microscope. The mRFP-Vpr localized in the nucleus, as reported previously [12], suggesting its properties were the same as those of the intact molecule. The mRFP-Vpr was then purified using ANTI-FLAG M2 agarose and run on sodium dodecyl sulfate-polyacrylamide gel electrophoresis (SDS-PAGE). The purified mRFP-Vpr was clearly detected as single band with an apparent molecular mass of approximately 50 kDa, which is consistent with the predicted amino acid sequence (Fig 2A). Purified mRFP-Vpr was then passed over the derivative-fixed PGS surface and binding was analyzed by SPR. As shown in Fig 2B, mRFP-Vpr specifically bound to the hematoxylin derivative.

### Ability of the hematoxylin derivativee to inhibit HIV-1 replication in macrophages

**Fig 3:**
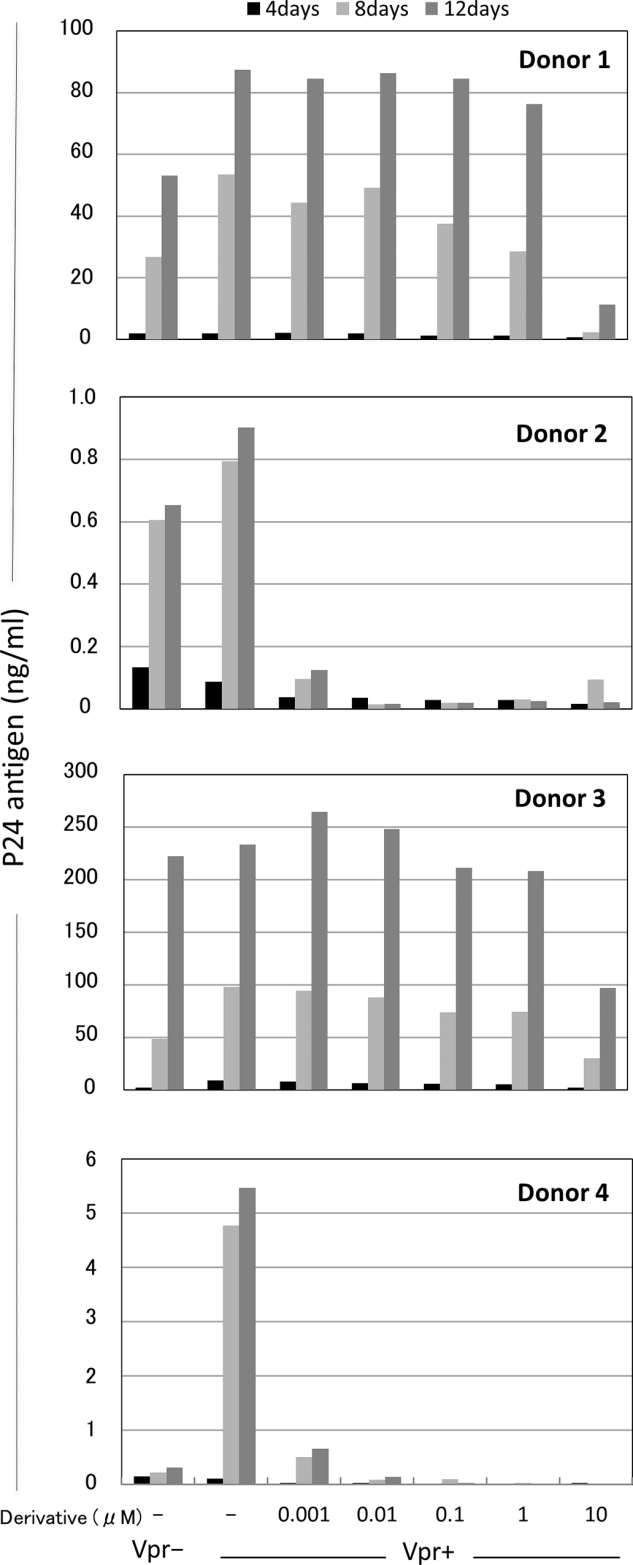
Derivative-mediated inhibition of viral replication in macrophages. Terminally differentiated primary macrophages (2×10^5^ cells/well) derived from four healthy donors were infected with HIV-1 (a total of 1 ng of p24) and then incubated with serial 10-fold dilutions of derivative (concentration range, 0 to 10 μM). The levels of virus in the culture supernatants were measured at 4, 8, and 12 days after inoculation in a p24 antigen ELISA. Data represent the mean p24 value from two wells.

To examine the ability of the hematoxylin derivative to inhibit HIV-1 replication, macrophages were isolated from ten healthy donors and these infectivities were tested by inoculation of macrophage-tropic pNF462 HIV-1 Vpr^+^ or Vpr^-^ virus. The infection of Vpr^+^ virus showed high p24 values compared with Vpr^-^ virus on macrophages from nine among ten donors and the infectivity on macrophages was much different dependent on the individual differences on donors (S1 Fig). Then, we selected macrophages derived from four donors and subsequently infected with the macrophage-tropic pNF462 HIV-1 virus. Primary macrophages were cultured in the presence of serial (10-fold) dilutions of the hematoxylin derivative (0 to 10 μM). After 4, 8, and 12 days of infection, viral replication was assayed in a p24 enzyme-linked immunosorbent assay (ELISA). All macrophages infected with the Vpr^+^ virus showed higher p24 values than those infected with the Vpr^-^ virus after 8 and 12 days post-infection (Fig 3). Our previous studies using cells isolated from more than ten donors showed similar results [12, 13]. The effective concentration that blocked HIV-1 infection by 50% (EC_50_) of the hematoxylin derivative was less than 1 nM when tested against macrophages derived from donors 2 and 4; however, the EC_50_ against macrophages derived from donors 1 and 3 ranged from 1.6–8.4 μM (Fig 3 and Table 1). The inhibitory effect of the hematoxylin derivative was donor-dependent, although it was only slightly better than that of hematoxylin in donor 1 (Table 1). The hematoxylin derivative had no effect on host cell morphology, and the 50% cytotoxic concentration (CC_50_) against Molt-4 cells was 51.4 μM (Table 1). Taken together, these results suggest that the hematoxylin derivative is stable and inhibits HIV-1 replication to a similar extent as the parent compound.

**Table 1:** Ability of compounds to inhibit HIV-1 replication.

		EC_50_(μM)^a^				CC_50_(μM)^b^
Compound	days	Donor 1	Donor 2	Donor 3	Donor 4	Molt4 cells
Hematoxylin	4 days	>10	<0.001	NT^c^	NT	40
	8 days	6.7	<0.001	NT	NT	
	12 days	7.0	<0.001	NT	NT	
Derivative	4 days	3.4	<0.001	3.9	<0.001	51.4
	8 days	1.6	<0.001	6.2	<0.001	
	12 days	5.5	<0.001	8.4	<0.001	

^a^Terminally-differentiated primary macrophages derived from four healthy donors were infected by HIV-1 and incubated with serial 10-fold dilutions of compounds (0 to 10 μM). The amount of virus in the culture supernatants was measured in a p24 antigen ELISA at 4, 8 and 12 days after inoculation. The EC_50_ of compounds after 4, 8, and 12 days of infection is shown.

^b^The CC_50_ of the compounds was determined in an MTT assay. Molt-4 cells (1 ×10^5^ cells/well) were cultured RPMI1640 containing serially-diluted compounds (0 to 100 μM) in 24 well plates for 2 days.

^c^NT: not tested

### Ability of the hematoxylin derivative to inhibit Vpr function

**Fig 4:**
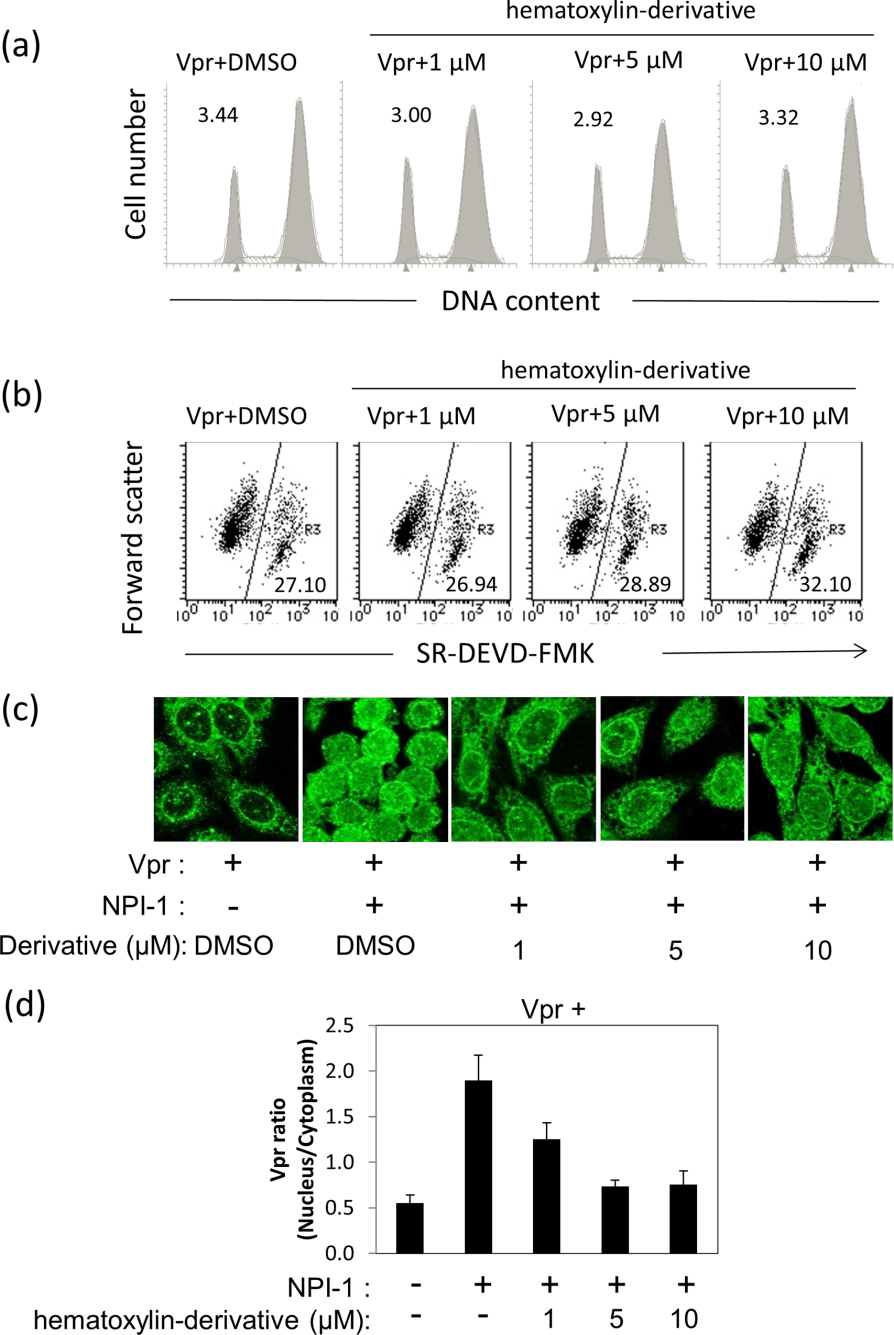
Effect of the derivative on Vpr function. (a) Effect of the derivative on cell cycle progression. HeLa cells (2.0×10^5^ cells) were harvested after 48 h incubation in the presence of 1, 5, or 10 μM of derivative or DMSO, and cell cycle profiles analyzed by flow cytometry. The G2/M:G1 ratios were calculated using ModFit LT Software (Verity Software House) and are shown in the upper right corner of each panel. (b) The effect of the derivative on apoptosis. HeLa cells (1×10^5^ cells) were cultured for 48 h in the presence of 1, 5, or 10 μM derivative or DMSO, and then stained with SR-DEVD-FMK to identify cells expressing active caspase-3. The percentage of cells expressing active caspase-3 was measured by flow cytometry (shown in the lower right corner of each panel). (c) Inhibition of Vpr nuclear transport. HeLa cells (2×10^6^) were seeded into an eight-well coverslip placed in a 10 cm dish. After 24 h of culture, cells were permeabilized with digitonin as described previously [10]. Digitonin-permeabilized HeLa cells were incubated with GST-Vpr (N17C74)-GFP in the presence of 1, 5, or 10 μM of derivative or DMSO and the GST-importin α isoform NPI-1, as described previously [24]. Cells were then analyzed under a confocal laser-scanning microscope (FV 1000; Olympus). (d) Efficiency of Vpr nuclear import was calculated by random measurement of Vpr fluorescence intensity in three small regions of interest (each 8.6 μm^2^) in the nucleus or cytoplasm. The mean fluorescence intensity and the ratio of nuclear-to-cytoplasmic Vpr in individual cells were calculated. Ten cells from each sample in C were randomly selected and the mean nuclear-to-cytoplasmic Vpr value was plotted. Error bars represent the SD of the nuclear-to-cytoplasmic Vpr ratio in ten individual cells derived from each sample.

Vpr has multiple functions, including the induction of G2/M arrest [14] and apoptosis [15, 16], nuclear import [6–9], transcriptional activation [17], and mRNA splicing [18, 19]. Previously, we reported that hematoxylin is a potent inhibitor of importin α-mediated nuclear import of Vpr [11]. In addition, the induction of G2 arrest by Vpr plays a particularly important role in efficient viral replication because the transcriptional activity of the HIV-1 long terminal repeat is most active during G2 phase [17, 20]. Vpr-mediated regulation of apoptosis is also important for immune suppression and pathogenesis during HIV infection [16, 21]. Therefore, we next examined the effect of the derivative on the major functions of Vpr during G2 arrest, apoptosis, and nuclear import. As shown in Fig 4A and 4B, the hematoxylin derivative had no effect on Vpr-induced G2 arrest and apoptosis. Next, we examined the ability of the hematoxylin derivative to inhibit the nuclear transport of Vpr in an *in vitro* nuclear import assay based on digitonin-permeabilized HeLa cells. For this, we used glutathione S-transferase (GST)- or green fluorescent protein (GFP)-tagged Vpr (N17C74) and recombinant importin α (isoform NPI-1). The hematoxylin derivative inhibited importin α-mediated nuclear import of Vpr, suggesting that it is a potent small-molecule inhibitor of Vpr nuclear entry (Fig 4C). However, the inhibitory effect of the hematoxylin derivativee reached a plateau at 5 μM (Fig 4D).

### Determination of the binding domain of Vpr using photoaffinity-linker-coated beads

**Fig 5:**
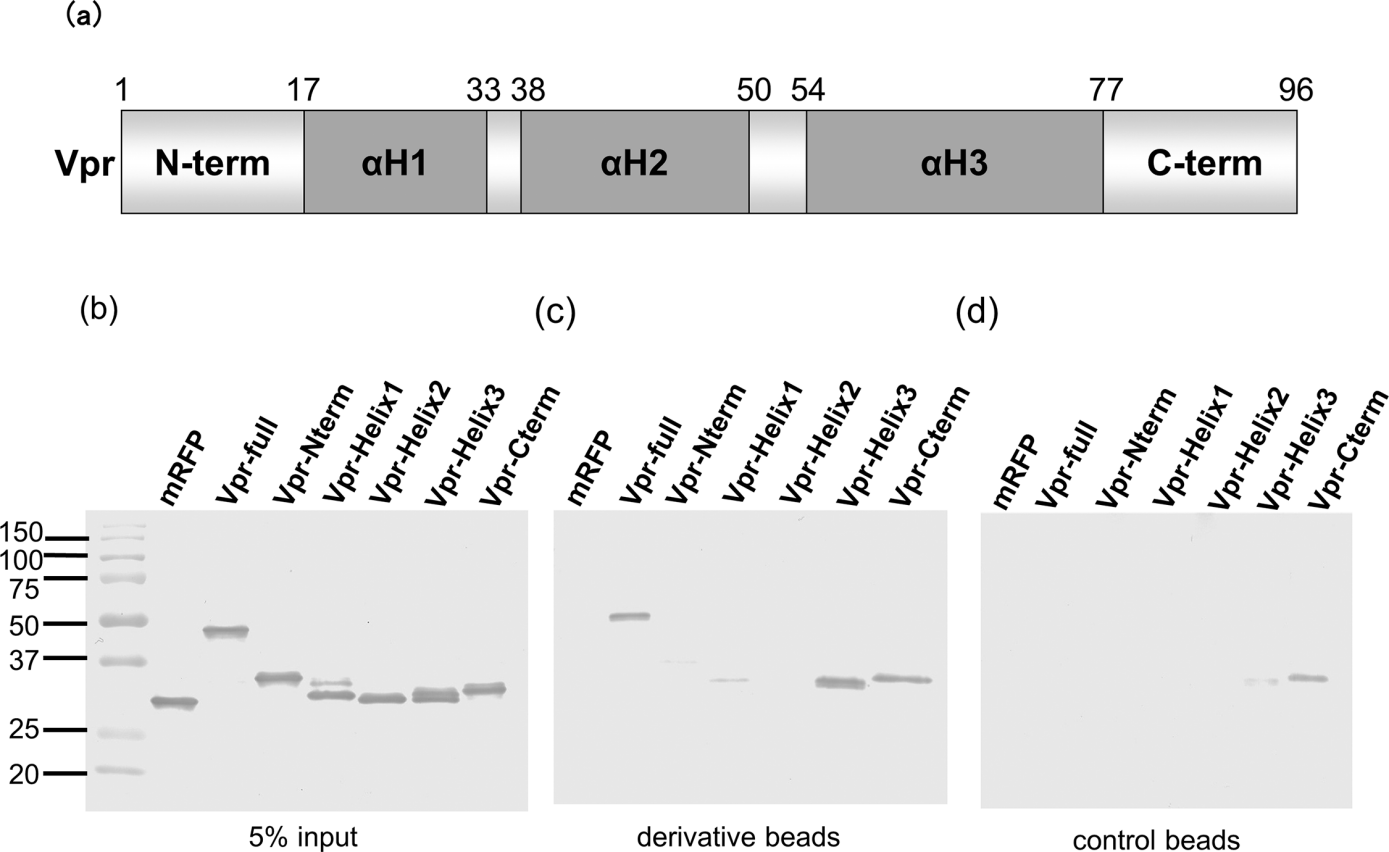
Use of a photo-cross-linked small-molecule affinity matrix to analyze the binding of Vpr to the derivative. (a) Schematic representation of the putative Vpr domains. NMR analysis revealed that full-length Vpr forms three amphipathic alpha helices, αH1, αH2, and αH3, which surround a hydrophobic core. Vpr has a flexible, negatively-charged N-terminal region flanking the helices, and its C-terminal region is also flexible, positively charged, and rich in arginine residues. (b–d) The derivative was cross-linked to Sepharose beads, which were then incubated with purified proteins at 4°C for 16 h. The proteins that bound to the derivative were separated on 15% SDS-PAGE and then detected by immunoblot analysis with an anti-FLAG M2 MAb (SIGMA). Bound proteins were detected with an anti-mouse IgG-alkaline phosphatase antibody (SIGMA). (b) Five percent of the total input protein was analyzed. (c, d) Proteins that bound to derivative cross-linked beads (c) and to control Sepharose beads (d).

As shown in Fig 5A, Vpr contains three well-defined α-helices, αH1, αH2, and αH3, which are surrounded by flexible amino-terminal and carboxy-terminal domains [22]. To identify the domain to which the hematoxylin derivative binds, we constructed expression plasmids containing each domain fused to FLAG-mRFP and transfected them into COS-7 cells. Each expressed protein was soluble and easily purified using ANTI-FLAG agarose, and yielded single bands with apparent molecular masses consistent with the predicted sequences (Fig 5B). These results suggest that the expressed Vpr domain assumes its native form. Next, we performed a binding assay using the purified chimeric proteins. Briefly, the proteins were incubated with the hematoxylin derivative (cross-linked to Sepharose beads) for 16 h at 4°C. After centrifugation, the proteins bound to the hematoxylin derivative were separated on 15% SDS-PAGE and then immunoblotted with an anti-FLAG M2 monoclonal antibody (MAb). As shown in Fig 5C, the hematoxylin derivative specifically bound to the full-length Vpr protein (Vpr-full) and to the αH3 domain. Weak binding to the αH1 domain was also detected. The C-terminal region bound to both hematoxylin derivative-beads and control beads, suggesting non-specific binding. These results suggest that the hematoxylin derivative mainly binds to the αH3 domain of Vpr (although it also binds weakly to αH1).

### Analysis of the highly conserved region within Vpr using the Wu-Kabat method

**Fig 6:**
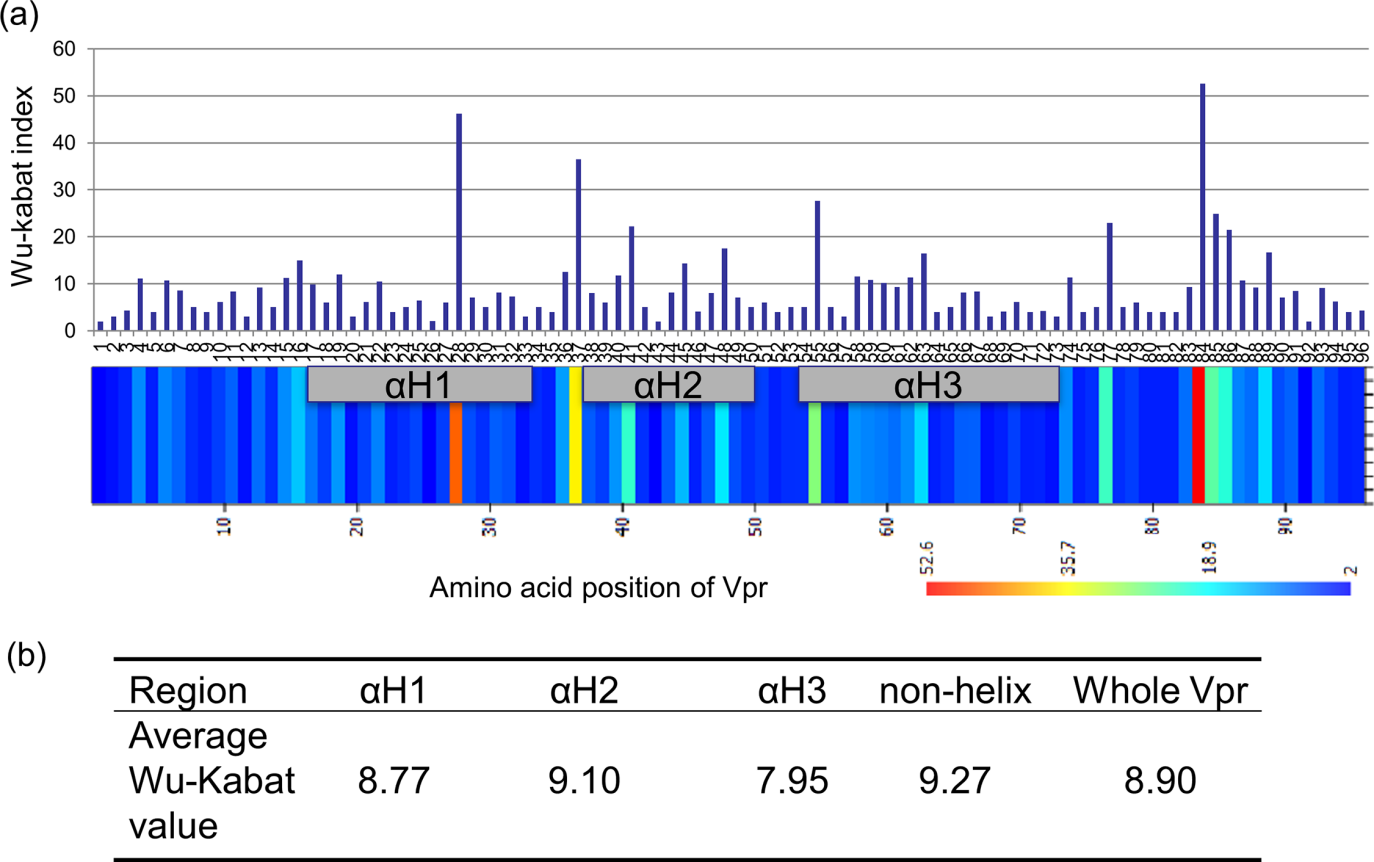
Analysis of the conserved regions within Vpr using the Wu-Kabat method. In total, 2,004 Vpr sequences were obtained from the HIV sequence database and used to calculate the Wu-Kabat index for each amino acid residue (a). The four Vpr regions were α Helix 1 domain (αH1, residues 17 to 33), α Helix 2 domain (αH2, residues 38 to 50), α Helix 3 domain (αH3, residues 54 to 74), and the non-Helix region (residues 1 to 16, residues 34 to 37, residues 51 to 53, and residues 75 to 96). These four regions plus the whole Vpr region (residues 1 to 96) were used to estimate the average Wu-Kabat index value (b).

Next, we identified the conserved region within Vpr according to the Wu-Kabat index, which was calculated using 2,004 HIV-1 Vpr sequences registered in the HIV sequence database in 2013 (http://www.hiv.lanl.gov/content/sequence/HIV/mainpage.html) (Fig 6A). We also calculated the average Wu-Kabat index value for each of the three helix regions and compared the values with those for the non-helix regions and for the whole Vpr region (Fig 6B). The Wu-Kabat value for the αH3 region (average index, 7.95) was much lower than that for the other regions [αH1 (average index, 8.77), αH2 (average index, 9.10), non-helix region (average index, 9.27), and the entire Vpr region (average index, 8.90)]. Therefore, αH3 is the most conserved region within HIV-1 Vpr.

## Discussion

Previously, we reported a novel anti-HIV-1 inhibitor, hematoxylin, which inhibits the nuclear import of Vpr by interfering with the interaction between Vpr and importin α [11]; however, hematoxylin is unstable upon exposure to UV light. Here, we generated a novel derivative of hematoxylin that is stable upon exposure to UV. The hematoxylin derivative contained three fewer aromatic hydroxyl groups than hematoxylin; however, it still inhibited both the importin α-mediated nuclear import of Vpr and HIV-1 replication in macrophages. The results of a macrophage-based HIV-1 replication assay revealed that the ability of the hematoxylin derivative to inhibit HIV-replication in macrophages derived from four healthy donors differed. Inhibitory activity was markedly higher in cells from donors with a low p24 value (donors 2 and 4) than in donors with high p24 value (donors 1 and 3). This suggests that the hematoxylin derivative is more effective in patients with a low viral load. Similar results were observed in our previous study. The hematoxyline derivative 16 showed inhibition activity at the concentration less than 10 nM in two among three donors. Macrophages derived from these donors also showed low p24 values less than 5 ng/ml [13]. These differences in efficacy may be due to differing expression of host factors, such as PRMT5, that bind directly to Vpr. Indeed, the level of PRMT5 expression alters the viral load in macrophages (Murakami et al., 2015, submitted).

The hematoxylin derivative inhibited HIV-1 replication in macrophages from donors 1 and 3 at a concentration of 10 μM (Fig 3); however, it showed no cytotoxic effects against Molt-4 cells at this concentration (CC_50_ = 51.4 μM; Table 1). Thus, the hematoxylin derivative specifically inhibits HIV-1 replication without exerting a cytotoxic effect against macrophages. Although we were able to alter the stability of the hematoxylin derivative, we could not alter its inhibitory activity. Future studies should examine the SAR of the hematoxylin derivative with a view to increasing its inhibitory activity so that it can be used at lower concentrations, making it suitable for administration to a wide range of patients with differing viral loads.

Because Vpr is a multifunctional protein, we also examined its ability to inhibit different Vpr functions, including induction of cell cycle arrest, apoptosis, and nuclear import. The hematoxylin derivative had no effect on Vpr-induced G2 arrest and apoptosis as well as hematoxylin. Alterations at the carboxyl terminus of Vpr (Arg 73) ablate both the transcriptional activity of Vpr and its ability to block cell cycle events at G2 [23]. Thus, Arg 73 is important for Vpr function. Although the binding domain of the hematoxylin derivative includes this residue, it did not inhibit Vpr-induced G2 arrest (Fig 4A). Thus, the binding of the hematoxylin derivative may be dependent upon another residue(s). However, the hematoxylin derivative inhibited importin α-mediated nuclear import of Vpr as efficiently as hematoxylin. Taken together, these results suggest that the derivative has similar properties to hematoxylin, but has a major advantage: it is more stable. Importantly, real-time PCR analysis showed that hematoxylin specifically inhibits nuclear import of the PIC [11], whereas the present study shows that the hematoxylin derivative specifically binds to Vpr to inhibit nuclear import *in vitro*. Thus, the hematoxylin derivative may inhibit HIV-1 replication in macrophages *in vivo* by inhibiting the nuclear transport of the PIC.

Vpr comprises five domains: an N-terminal region, αH1, αH2, αH3, and a C-terminal region. Therefore, we next identified which of these domains was the target for the hematoxylin derivative. We found that the hematoxylin derivative mainly bound to αH3 (although weak binding to αH1 was also detected). It appears that Vpr is first targeted to the nuclear envelope via its interaction with αH3. It then enters the nucleus via the interaction between the αH1 and importin α. We previously showed that the interaction between αH1 and importin α is important not only for nuclear import of Vpr but also for HIV-1 replication in macrophages [7]. In addition, the interaction between the nuclear envelope and αH3 appears to be essential for HIV-1 replication in macrophages (data not shown). Thus, the hematoxylin derivative interacts with domains αH1 and αH3, and then directly inhibits the binding of Vpr to importin α and/or the interaction between Vpr and the nuclear envelope. It does this without disrupting the common structure of Vpr because it does not inhibit Vpr-mediated G2 arrest or apoptosis. Vpr adopts an antiparallel dimer structure via αH3. The interaction between His71 and Trp54 within αH3 of Vpr increases the thermal stability of the antiparallel helical dimer, and the helix arrangement generates a more compact structure [24]. Nuclear magnetic resonance analysis shows that the helix-turn-helix structure of Vpr places the αH1 and αH3 domains in close proximity to each other [22]. We also postulate that the hematoxylin derivative binds to αH1 and αH3, and that the strong interaction with αH3 alters the folding structure of the Vpr protein, thereby inhibiting its interaction with importin α. Notably, the amino acid sequence of Vpr is more conserved than that of other viral proteins such as integrase, protease, or reverse transcriptase. In particular, Wu-Kabat analysis showed that the αH3 domain was highly conserved, suggesting that such a domain is a good target for the development of an antiviral agent.

## Conclusions

In conclusion, we succeeded in generating a stable HIV-1 inhibitor that binds to the αH3 domain of Vpr and blocks its nuclear import in macrophages, thereby inhibiting HIV replication. We previously identified a Vpr-binding compound that interacted directly with purified full-length Vpr and inhibited HIV-1 replication in macrophages [12]. Viral accessory proteins are not essential for HIV-1 replication, although they do play an important role; thus the inhibitory region of Vpr (which is highly conserved) is a potential therapeutic target for reducing the viral load in cases of latent macrophage infection or drug-resistant HIV-1 infection. At this moment in time, several steps in the HIV-1 replication cycle (e.g., viral adsorption, entry, cell fusion, integration, viral mRNA transcription, nuclear import of the viral genome, assembly, and budding and maturation into infectious particles) are potential targets for new drugs. The results reported herein demonstrate that Vpr is one such target and could be used as a basis for developing compounds capable of inhibiting HIV-1 replication.

## Materials and Methods

### Ethics statement

Blood samples were provided from human healthy donors, all of whom provided written informed consent. All experiments in this study were approved by the RIKEN Ethics Committee [Certificate no. Wako 21-2(3)].

### Synthesis of a stable hematoxylin derivative

2,2-Dimethoxypropane (2.18 g, 21.0 mmol), p-toluene sulphonic acid (200 mg, 1.16 mmol), and phosphorus pentoxide (3.14 g, 22.1 mol) were added to a solution of hematoxylin (6.08 g, 20.1 mmol) in dry acetone (100 mL) and the mixture was refluxed for 12 h. Then, phosphorus pentoxide (10.0 g, 70.4 mmol) was added and the mixture was refluxed for an additional 10 h. After cooling, the reaction mixture was quenched with ice-water and the aqueous phase was extracted with ethyl acetate. The organic layer was washed with brine, dried over Na_2_SO_4_, and then concentrated. The crude product was purified by flush chromatography (hexane/ethyl acetate [2:1]) to yield 1.91 g (28%) of acetonide-protected hematoxylin. Next, allyl bromide (0.10 mL, 1.2 mmol) and potassium carbonate (107 mg, 0.750 mmol) were added to a solution containing acetonide-protected hematoxylin (178 mg, 0.521 mmol) in dry dimethylformamide (4.0 mL) and the reaction mixture was stirred for 20 h at room temperature. The reaction mixture was then poured into water and the mixture was extracted with ethyl acetate. The organic layer was washed with brine, dried over Na_2_SO_4_, and then concentrated. Finally, the crude product was purified by flush chromatography (hexane/ethyl acetate [2:1]) to yield 44.1 mg (22%) of derivative.

### Cell culture

COS-7, 293T, HeLa, and Molt-4 cells were cultured in Dulbecco’s modified Eagle’s medium (SIGMA) or RPMI 1640 (Invitrogen) containing penicillin, streptomycin, glutamine (GIBCO), and 10% fetal bovine serum (SIGMA).

### Construction of expression plasmids

pCAGGS vectors encoding FLAG-mRFP full-length Vpr (Vpr-full) or FLAG-mRFP (mRFP) and pGEX-6P-3 vectors encoding GST-mutant forms of Vpr N17C74-green fluorescent protein (GFP) (N17C74) or GST-tagged-NPI-1(NPI-1) have been described previously [12, 25, 26]. The FLAG-mRFP-N-terminal region (N), FLAG-mRFP-αH1, FLAG-mRFP-αH2, FLAG-mRFP-αH3, or the FLAG-mRFP-C-terminal region (C) were amplified by PCR and then cloned into pCAGGS. The predicted amino acid sequence of Vpr-full was identical to the sequence previously reported for pNL432 [27] (GenBank ID: M19921).

### Expression and purification of proteins

COS-7 cells (1 × 10^6^) were transfected with 10 μg of pCAGGS vector encoding Vpr-full, N, αH1, αH2, αH3, C, or mRFP using the FuGene HD Transfection Reagent (Roche). Two days later, the expressed proteins were purified using ANTI-FLAG M2 agarose (SIGMA), as described previously [12, 28].

The pGEX-6P-3 vectors encoding N17C74 or NPI-1 were expressed in *Escherichia coli* strain BL21 CodonPlus (DE3)-RIL (Stratagene) and purified as described elsewhere [26].

### SDS-PAGE and Immunoblot analysis

Purified proteins were separated on 15% SDS-PAGE under reducing conditions and then stained with Coomassie brilliant blue. Immunoblot analysis was performed using standard methods, as described previously [12, 28], with an anti-Flag MAb, followed by incubation with an anti-mouse IgG-alkaline phosphatase antibody (SIGMA).

### SPR analysis to detect binding of Vpr to the derivative

PGS was prepared as described previously [29, 30]. Solutions of the test compounds were spotted onto a PGS-coated chip and irradiated (4 J/cm^2^) at 365 nm in a CL-1000L ultraviolet cross-linker (UVP Inc.) under a UV transmission filter (Sigma-Koki).

The compound-immobilized PGS chip was then placed in a MultiSprinter SPR imaging instrument (Toyobo) and mRFP or mRFP-Vpr was injected onto the PGS surface at a flow rate of 0.1 mL/min. SPR images were constructed using the Scion Image program (Scion).

### Macrophage preparation

Human peripheral blood mononuclear cells (PBMCs) were collected from four healthy donors and isolated on a Ficoll gradient (Immuno-Biological Laboratories). Monocytes were isolated from PBMCs using MACS CD14 MicroBeads, a MACS Separation column, and a QuadroMACS Separation Unit (Miltenyi Biotec), as previously described [7]. Monocytes were cultured for 10 days in RPMI 1640 containing 10% fetal bovine serumn, 5% AB serum, and human macrophage-colony stimulating factor (10 ng/mL; PeproTech EC) to promote differentiation into mature macrophages.

### Viral infection and determination of the viral replication inhibitory effect in macrophages

HIV-1 was produced by transfecting 293T cells with macrophage-tropic pNF462 proviral DNA encoding either wild-type Vpr [31] or a deficient form of the Vpr protein [25]. The relative p24 values were determined by titrating the viral stocks using an in-house ELISA, as described previously [32]. Macrophages (2×10^5^ cells/well) were infected with HIV-1 (a total of 1 ng of p24) in the presence of serial 10-fold dilutions of the different test compounds (0 to 10 μM). Cell supernatants were collected 4, 8, and 12 days post-infection and the p24 levels were measured in an ELISA. The antiviral activity of the compounds was based on the EC_50_.

### 3-(4, 5-dimethylthylthiazol-2-yl)-2, 5-diphenyltetrazolium bromide (MTT) assay and cell cycle and apoptosis analyses

Cell viability, cell cycle status, and caspase-3 activation were examined in MTT, flow cytometry, and SR-DEVD-FMK (ImmunoChemistry Technologies) assays, respectively, as described previously [12, 14].

### *In vitro* nuclear transport assay

Digitonin-permeabilized HeLa cells were incubated with GST-Vpr (N17C74)-GFP and the GST-importin α isoform NPI-1, as described previously [10, 26]. Cells were then analyzed under a confocal laser-scanning microscope (FV 1000; Olympus).

### Photo-cross-linked small-molecule affinity matrix assay

The derivative was cross-linked to Sepharose beads as previously described [33]. A solution of the compound in methanol was added to the photoaffinity-linker-coated beads, and the mixture was concentrated and dried *in vacuo*. The beads were then irradiated at 365 nm (4 J/cm^2^) in a UV cross-linker and washed with methanol to yield compound-cross-linked affinity beads.

Each of the recombinant proteins was then incubated with the compound-cross-linked affinity beads and the proteins that bound to the compound were then separated on 15% SDS-PAGE followed by immunoblot analysis with an anti-FLAG M2 MAb.

### Wu-Kabat analysis of the conserved region within Vpr

The Wu-Kabat variability index is a well-established descriptor of the susceptibility of an amino acid position to evolutionary replacement and was calculated as described previously [34].

## Supporting Information

S1 Fig Infectivity of pNF462 HIV-1 Vpr+ or Vpr- virus on macrophages. (TIF)
