# Supplementary figures and images for: Synthesis of a Vpr-Binding Derivative for Use as a Novel HIV-1 Inhibitor
Source: PLoS One. 2015 Dec 23;10(12):e0145573. doi: 10.1371/journal.pone.0145573 (PMC4689350; doi:10.1371/journal.pone.0145573)

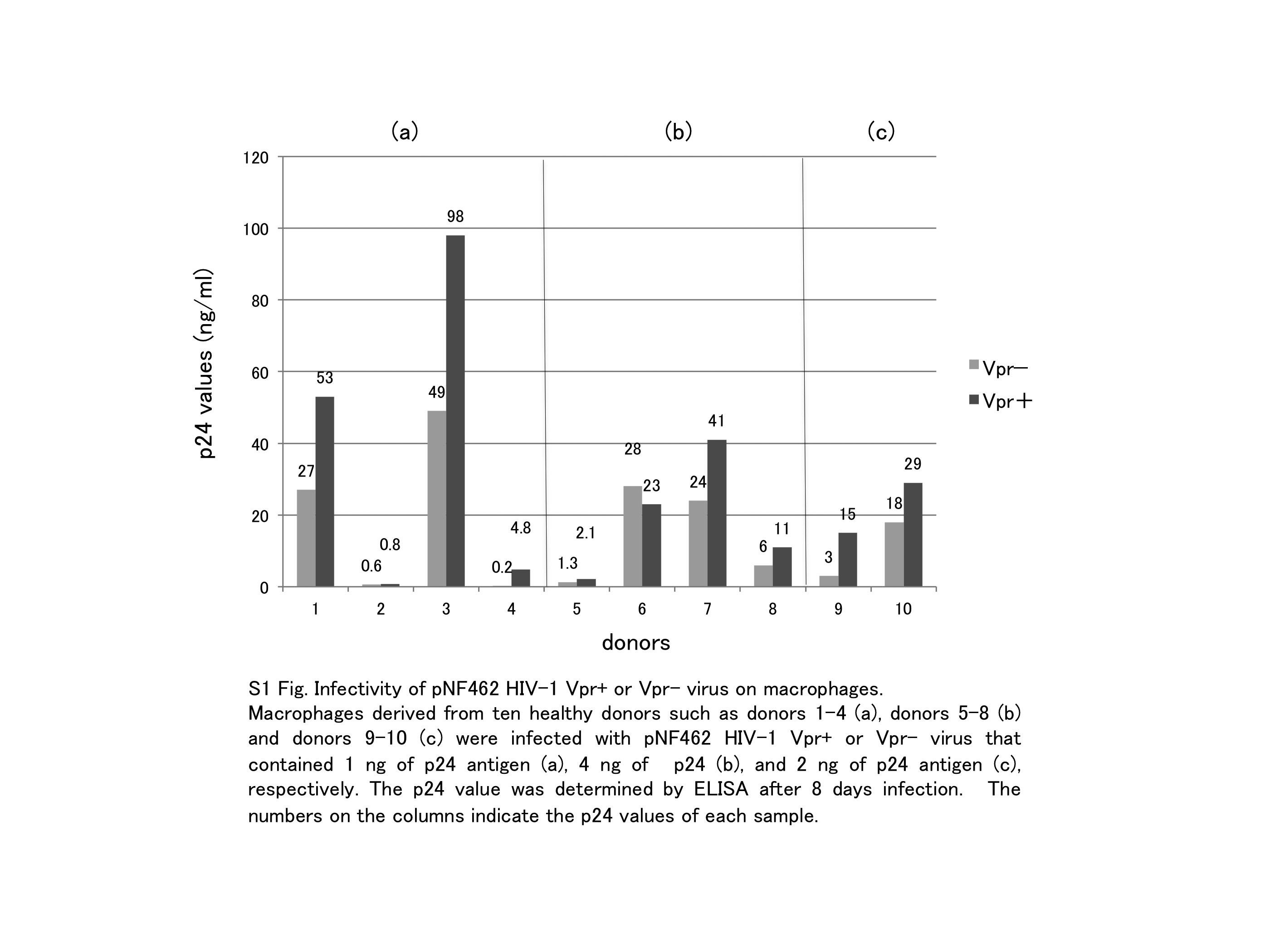

Supplement: S1 Fig — (TIF) [file pone.0145573.s001.tif]
